# Comparison of PIXE and XRF in the analysis of silver denarii of the early Piast

**DOI:** 10.1007/s10967-017-5556-8

**Published:** 2017-10-20

**Authors:** Janusz Lekki, Marta Matosz, Czesława Paluszkiewicz, Ewa Pięta, Tomasz Pieprzyca, Zbigniew Szklarz, Julio M. del Hoyo Meléndez

**Affiliations:** 1 Institute of Nuclear Physics, Polish Academy of Sciences PAS, Radzikowskiego 152, 31-342 Cracow, Poland; 2 Laboratory of Analysis and Non-Destructive Investigation of Heritage Objects, The National Museum in Krakow, 31109 Cracow, Poland

**Keywords:** Micro-PIXE, Micro-XRF, Silver denarii, Quantitative elemental composition, Medieval Poland, Numismatic collections

## Abstract

The collection of denarii from the time of development of a Polish medieval state was studied using the proton induced X-ray emission spectroscopy. The major elements detected for these denarii are Ag and Cu, while minor elements such as Pb, Fe, Au, Bi, and Zn may also be present. The aim of the study was to cross-compare the results with a previous micro-X-ray fluorescence data and to perform a better quantification of the denarii elemental composition, especially for trace elements, providing suggestions of the origin of alloy compounds.

**Electronic supplementary material:**

The online version of this article (doi:10.1007/s10967-017-5556-8) contains supplementary material, which is available to authorized users.

## Introduction

Little information is available about the chemical composition of the oldest Polish denarii. The period concerned includes the reign of the three first Polish rulers: Mieszko I (ruled 962–992), Boleslaus the Brave (992–1025) and Mieszko II Lambert (1025–1031). The collection housed at the National Museum in Cracow consists of 71 items, minted in the period of about 995 to 1020. The coins were made of a Ag–Cu alloy, with addition of several less abundant elements with Pb being the most significant.

In a previous study del Hoyo-Melendez and co-workers (2015) examined this collection using micro-X-ray fluorescence (XRF) spectrometry and multiple conclusions have been drawn, mostly based on the quantitative analysis of major elements. The current study deals with the use of particle induced X-ray emission (PIXE) spectroscopy to perform a cross-comparison of the results obtained with both analytical techniques. In addition, better quantification of trace elements is possible through the use of these two complementary techniques. The information concerning elemental content is interesting since it may provide evidence about the alloys, the metal sources, and the technological processes employed to produce the denarii. The main objective of the study was to provide analytical data that may serve to enhance the existing knowledge about the beginnings of the Polish state.

## Experimental

### Denarii

**Fig. 1:**
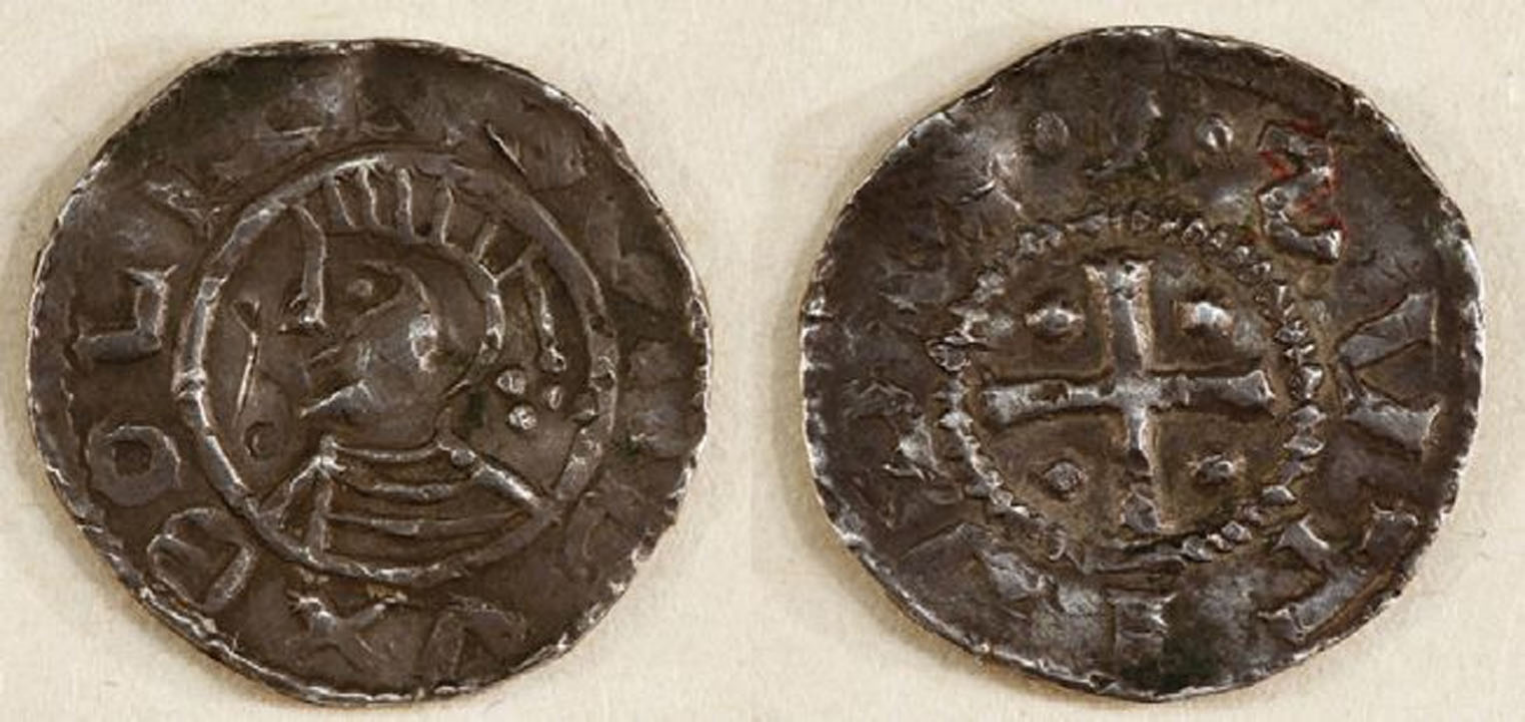
Obverse and reverse of the Boleslaus the Brave denar (image: Piotr Frączek)

A collection of 71 Ag denarii minted between 995 and 1020 AD, from the Numismatic Cabinet of the National Museum in Cracow was investigated. The evaluated objects are divided into two broad categories: Boleslaus the Brave and Mieszko II Lambert. A full historical and archaeological description of the denarii can be found elsewhere [1, 2]. An example of one of the evaluated denarii, namely Boleslaus Type XI.17 is presented in Fig. 1. Images on the left and right hand side correspond to obverse and reverse, respectively.

The physical characteristics of the denarii are as follows: weight: 0.5–3.0 g, thickness: 0.5–1.0 mm, diameter: 16–25 mm. Their size and good state of preservation make them ideal for investigations involving ion beam analytical techniques.

### Micro-X-ray fluorescence (XRF) spectrometry

Micro-XRF analyses were carried out using a Bruker (Karlsruhe, Germany) Artax 400 XRF spectrometer equipped with a Rh tube and a collimator capable of producing an analytical spot of about 0.650 mm. Bright and flat areas of the denarii were analyzed to avoid zones showing signs of corrosion and minimizing surface effects, respectively. The X–ray generator was operated at 50 kV and 0.5 mA, while the acquisition time was 300 s. This instrument has a Si drift X-ray detector with an active area of 10 mm^2^. The beam was focused on the analysis spot with the help of a laser and a camera, which are attached to the spectrometer. This instrument allows to perform non-destructive and non-contact measurements, while providing simultaneous multi-elemental analysis in the range from Na to U. The average relative error estimated after evaluating a series of Ag–Cu–Pb standards was Ag ~ 2%, Cu ~ 11%, and ~ 14% for Pb. Acquisition and evaluation of XRF spectra was carried out using Spectra 5.3 (Bruker AXS Microanalysis, Berlin, Germany). The methodology has been described elsewhere [3, 4].

### PIXE—technique and experimental details

The surface composition of the denarii was determined by means of micro-proton-induced X-ray emission (μPIXE), using the proton microprobe facility at the HVEC K-3000 Van de Graaff accelerator of the Institute of Nuclear Physics (INP) in Cracow. For measurements, samples were placed in the experimental chamber (vacuum ~ 10^−5^ hPa) and positioned in the microprobe focus using an on-line microscope for accurate selection of the investigated area. Additional details about the microprobe experimental setup have been described elsewhere [5].

The beam energy was 2.05 MeV and the beam current intensity was in the range of ~ 200 to 300 pA. Rectangular areas (256 × 256 µm^2^, 128 × 128 pixels) were scanned with a proton beam of ~ 20 µm in diameter. Due to the non-homogeneous surface characteristics of the denarii, scanning mode was applied, however mostly not to obtain elemental area maps, but to collect more reliable data averaged from larger areas.

Samples of the studied material were deposited on a surface of a conducting carbon adhesive tape and next fixed to a sample holder and placed in the vacuum experimental chamber. To minimize the surface geometry effects, the beam spot position was always selected to cover a relatively flat area. The exact knowledge of the bombarding beam current was gained in the procedure following the basic idea presented in [6], where, during the measurement, the beam was periodically deflected and directed to a Faraday cup with a current integrator, monitored by the data acquisition system of the microprobe. For typical scanning experiments, such arrangement enables a line by line normalization of the X-ray data (μPIXE map), but in our case it served only to determine the number of protons bombarding the sample (target charge). X-ray and proton backscattering spectra were collected in the event-by-event mode using the multiparameter data acquisition system of the microprobe. The typical collection time was less than 10 min, corresponding to a total beam charge of at least ~ 0.1 µC. The induced X-rays were registered by the Princeton Gamma Tech Si(Li) detector characterized by a resolution of 160 eV for the energy of 5.9 keV and an active area of 80 mm^2^. The detector was placed 25 mm from the irradiated sample and a 260 µm Kapton attenuation filter was placed between the detector and the sample. Data collection and processing was carried out using the INP microprobe proprietary software, interacting with two parallel systems: a digital and an analog one. The latter is based on the CAMAC system, while the former employs a XiA XMAP digital signal processor. The X-ray spectra collected by both systems were quantified using the GUPIX software package [7]. It is worth to mention that the outputs produced by both systems were close, but the digital one was delivering better performance and more reliable results. The GUPIX code provided a realistic estimation of errors, including the statistical part and the error related to the physical model and curve fitting. The typical relative error for matrix elements found in the studied denarii were as follows: Ag (Kα line) < 1%, Cu (Kα) < 1%, Pb (Kα) < 1%.

In both techniques, XRF and PIXE, the time of single measurement was less than 10 min. Due to differences in cross sections for X–ray production and the intensity of the bombarding flux, the XRF spectra were up to one magnitude higher. However, due to specificity of the method, only PIXE enables reliable quantification of less abundant elements, including trace elements. The theoretical detection limit of the PIXE method extends to single ppm. In the current study, trace elements were reliably quantified to concentration levels of few hundreds of ppm.

## Results and discussion

**Fig. 2:**
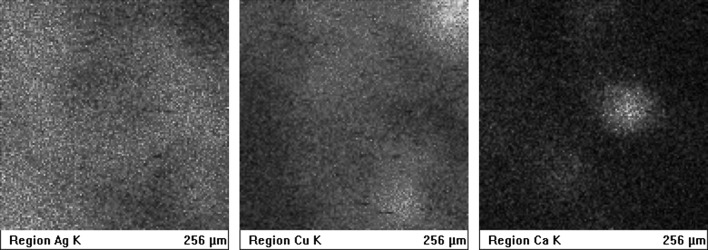
PIXE elemental maps obtained for major elements Ag and Cu, proving a non-uniform distribution of the X-ray signal from the denarius surface. The third map most probably shows an example of surface contamination with Ca

The following Fig. 2 shows variations in the aerial intensity of the characteristic X-ray signal, due to local contamination, inhomogeneity of the alloy (element segregation in silver-copper alloys), and surface corrosion. These factors force the requirement of multiple measurements of every single denar.

**Fig. 3:**
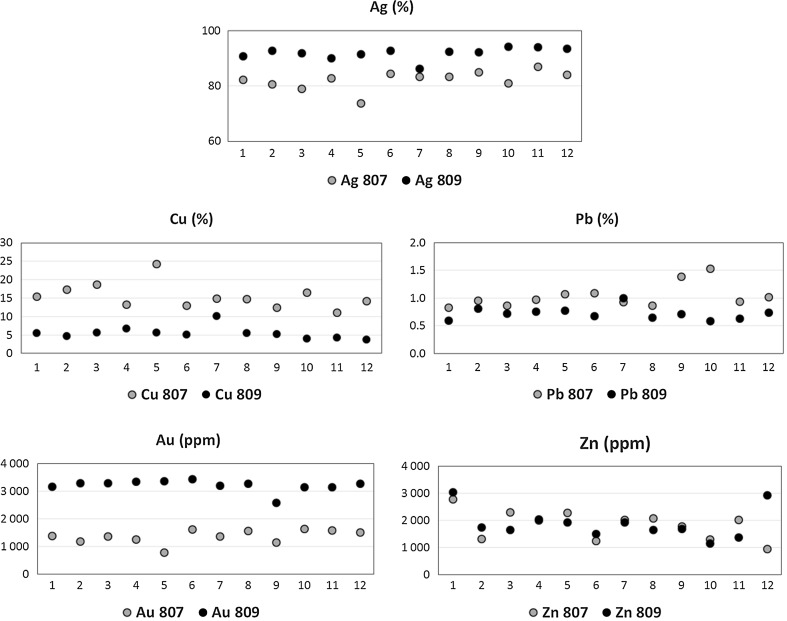
Elemental concentrations (weight) of the most abundant elements (Ag, Cu, Pb, Au, Zn), measured in 12 random positions at the surface of two denarii (coin inventory numbers 807 and 809)

As a reasonable compromise between accuracy and measurement time, typically three measurements of the obverse and three of the reverse were performed. To obtain a better estimation of the possible consequences of this compromise, several denarii were measured in 12 areas (instead of a standard number of 6). Figure 3 shows the results of these extra measurements on two denarii, illustrating limts of reproducibility of single measurements.

**Fig. 4:**
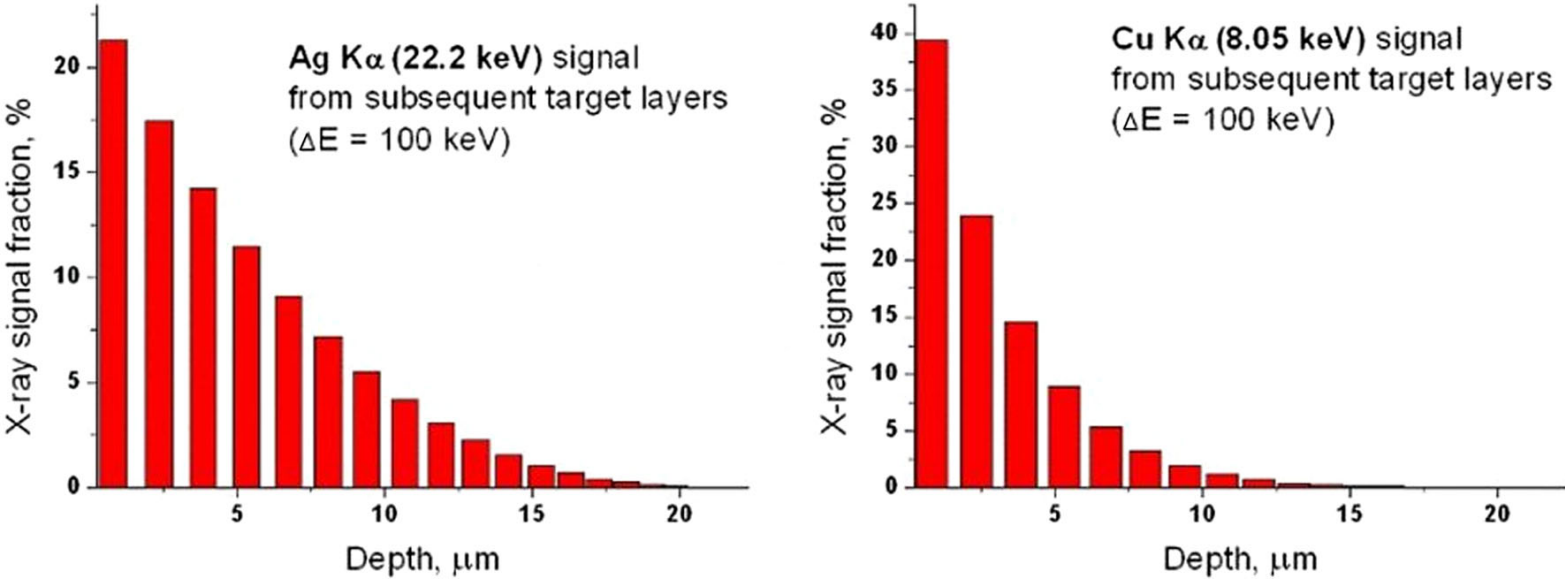
Fractions of the characteristic X–ray signal for the Ag and Cu K–alpha lines reaching the detector, computed as a function of X–ray origin depth

In these 12-elements sets, the standard deviation of a single measurement was approximately 2–3% for Ag, 15–17% for Cu, 11–21% for Pb, 7–12% for Au, and 30% for Zn. Major elements results are in reasonable agreement with the standard deviation of previous μXRF measurements [3]. These results show that also in the case of less abundant major elements, like Cu and Pb, extreme results of a single measurement of the same denar may differ by a factor of two. For even less abundant elements, like e.g. Zn, such effect is even more severe. This observation, together with data delivered by aerial elemental maps, sets the reliability limit for the analysis and discussion of the results. The very limiting factor, particularly significant in the case of PIXE, is the limited depth (or volume) from where a useful X–ray signal may be detected. Due to contamination effects on the denarii surface and silver enrichment of the upper denar layer [8, 9], it is usually difficult to reach the bulk of the studied object. The following Fig. 4 illustrates the fraction of the total X–ray signal registered in the detector versus the depth of the X–ray origin, computed for an example, perfect coin, assumed to contain 95% Ag and 5% Cu (stoichiometry). Stopping powers for 2.05 MeV protons penetrating the target have been calculated using the SRIM package [10], while the X–ray production data were delivered by the GUCSA code, being a part of the GUPIX software [7].

For the Ag–Cu denarii and the applied energy of protons (2.05 MeV), the maximum depth was about 12 µm for the characteristic radiation of Cu (Kα) and 17 µm for Ag (Kα). In XRF studies these values were higher (especially the value for Ag line, by a factor of about 2). However, the quantification method used in this technique (comparison with Ag–Cu standards), in case of layered structure of denarii, had even a more pronounced effect in the reliability of concentration results as compared to PIXE.

**Fig. 5:**
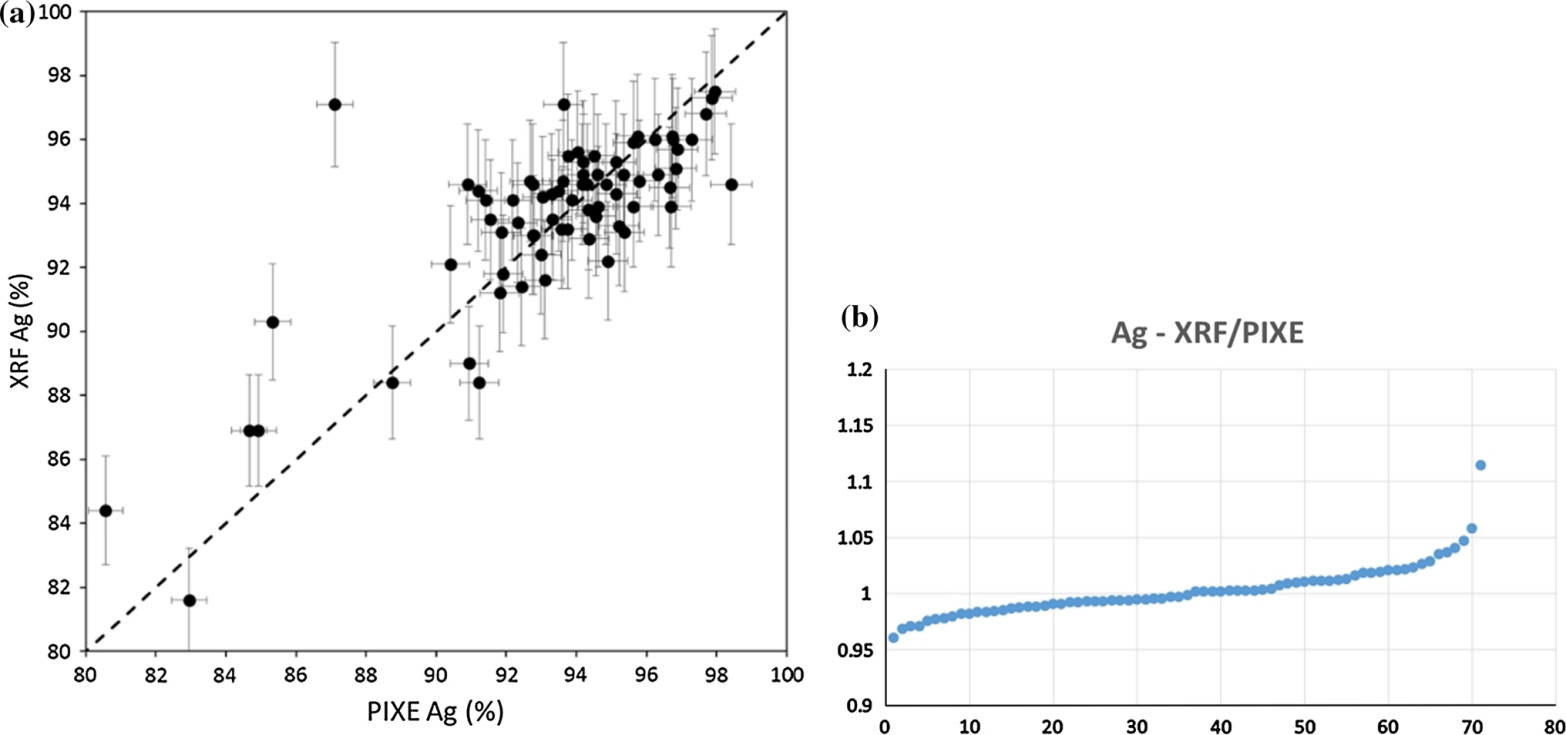
Ag content—comparison of all XRF and PIXE measurements: **a** XY plot, **b** ordered plot: relation between XRF and PIXE results plotted against denar number

**Fig. 6:**
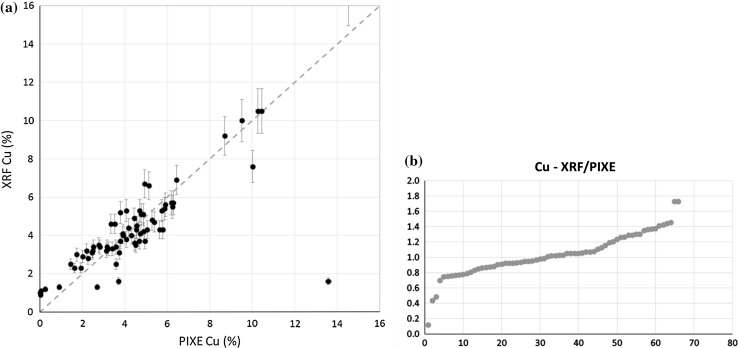
Cu content—comparison of all XRF and PIXE measurements: **a** XY plot, **b** ordered plot: relation between XRF and PIXE results plotted against denar number

Figures 5 and 6 summarize final results for the whole collection, illustrating the dependence between Ag and Cu content, determined by XRF and PIXE.

In the case of Ag, the obtained results show a good agreement between both experimental techniques, virtually all data are consistent and remain within a ± 5% range.

For Cu, the agreement between XRF and PIXE results is worse, relative to that obtained for Ag. Not only the range of results is broader, but also many excessive points are present. In particular, few denarii with a very low Cu content determined by PIXE (below 1000 ppm) are characterized by a high Ag content in the 97–98% range and less significantly, by the relative high Pb content of ~ 2%. This deficit of Cu could be caused by corrosion processes leading to silver surface enrichment. Disturbances in the surface layer, including Ag enrichment and deposition of corrosion products, are reflected in variations registered after evaluating the measurements of Ag K and Ag L lines intensities. As the L line is more prone to attenuation than the K one, the signal corresponding to the L line is limited to a depth of few microns, while the K line signal is collected from a larger depth. Therefore, excess of the Ag L line intensity in relation to the K line (as compared to the homogeneous Cu-Ag standard) suggests Ag enrichment (and thus Cu deficiency) in the near-surface layer. The opposite effect may be attributed to deposition of corrosion products in the near-surface layer.

The comparison of quantitative XRF and PIXE data has been restricted to the two major elements as the content of the next abundant element (Pb) can be reliably quantified only by PIXE (concentration mostly below 1%).

**Fig. 7:**
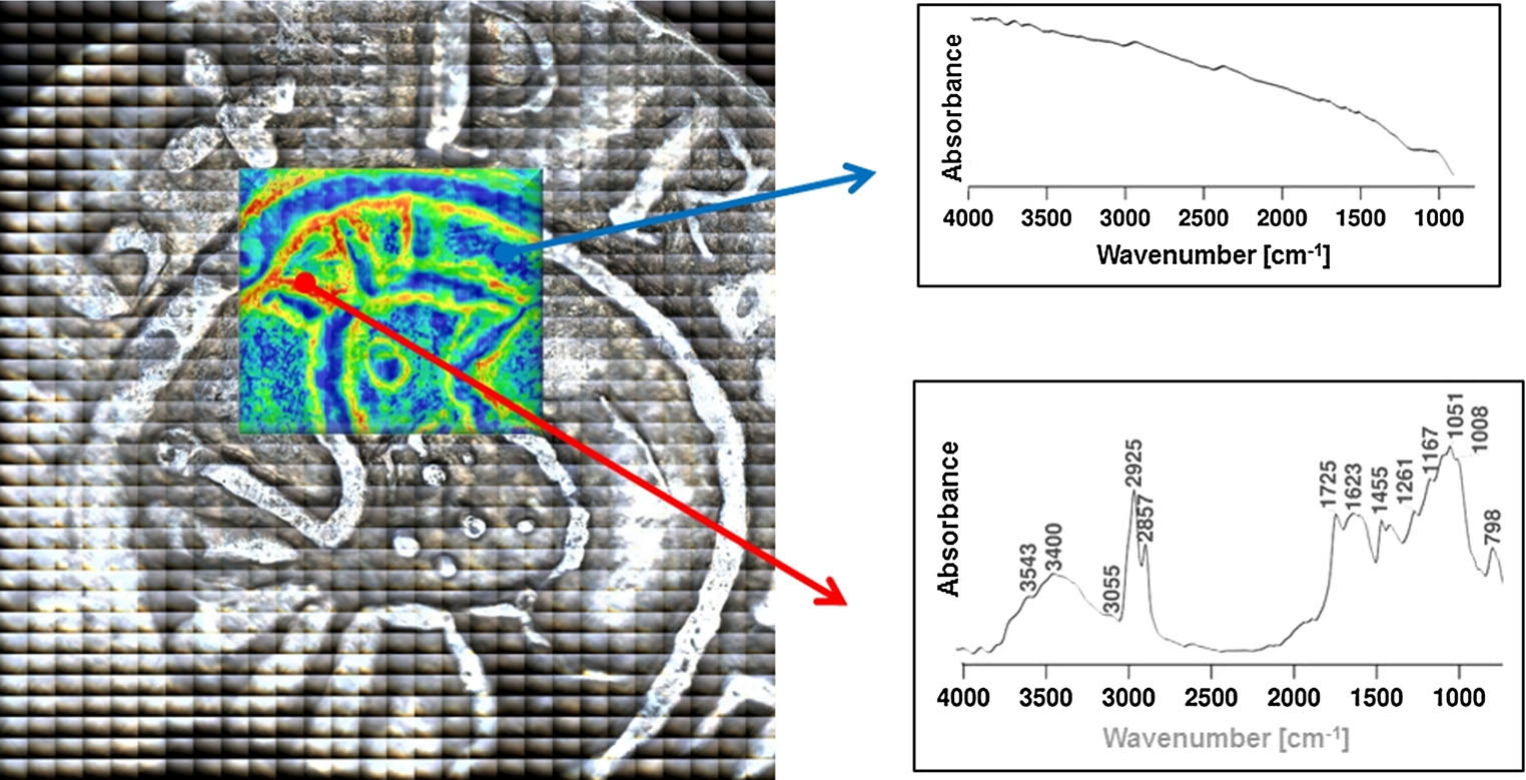
FTIR image mosaic of a Boleslaus the Brave denar with the exemplary spectra collected from two regions

Scattering of the results is enhanced by the requirement that the investigated objects could not be neither cleaned nor treated. As this was indeed the case for the Cracow collection, additional FTIR measurements were carried out in order to assess the contamination layer on the denarii surface (Pięta et al. forthcoming). Figure 7 shows two examples of FTIR spectra, corresponding to two different areas on the denar surface.

The differences in the spectra are extreme. The first spectrum contains almost no valuable data. In the second one, several, well defined bands can be identified: water (3543 and 3400 cm^−1^), lipids (2925, 2857, and 1725 cm^−1^), phosphates (1051 cm^−1^), and silicates (798 cm^−1^).

These experimental difficulties stress the importance of a complementary and multi-technique approach for these kinds of studies especially when the Ag-enriched layer can have a thickness greater than 100 µm showing the clear limitation of both techniques due to their low penetration depth. Apart of the methods described in the current paper, neutron activation analysis (NAA) has been explored as a potential technique for obtaining data delivered from the whole volume of the investigated object. Therefore, for these kinds of objects NAA may serve as a validation tool for XRF and PIXE techniques.

**Fig. 8:**
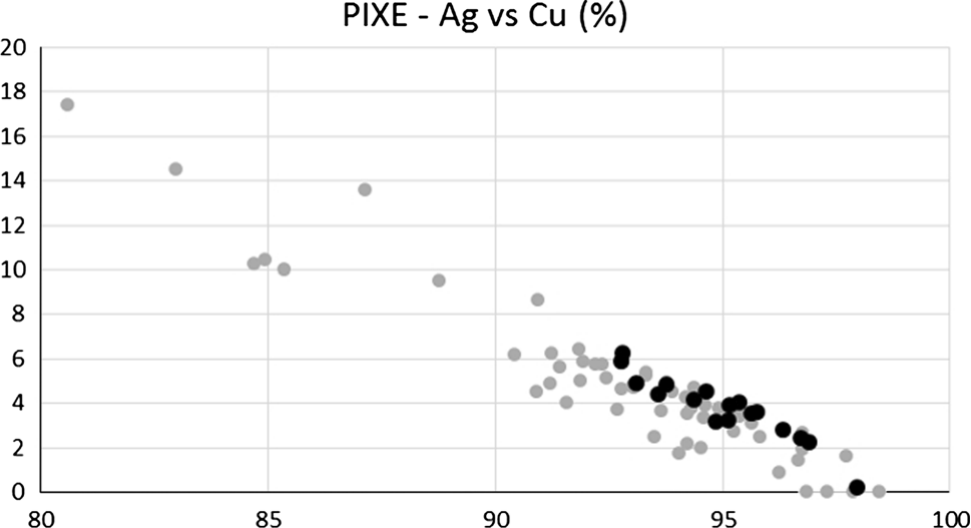
Ag (horizontal) versus Cu content. Black points: Mieszko II denarii, grey points: Boleslaus denarii

**Fig. 9:**
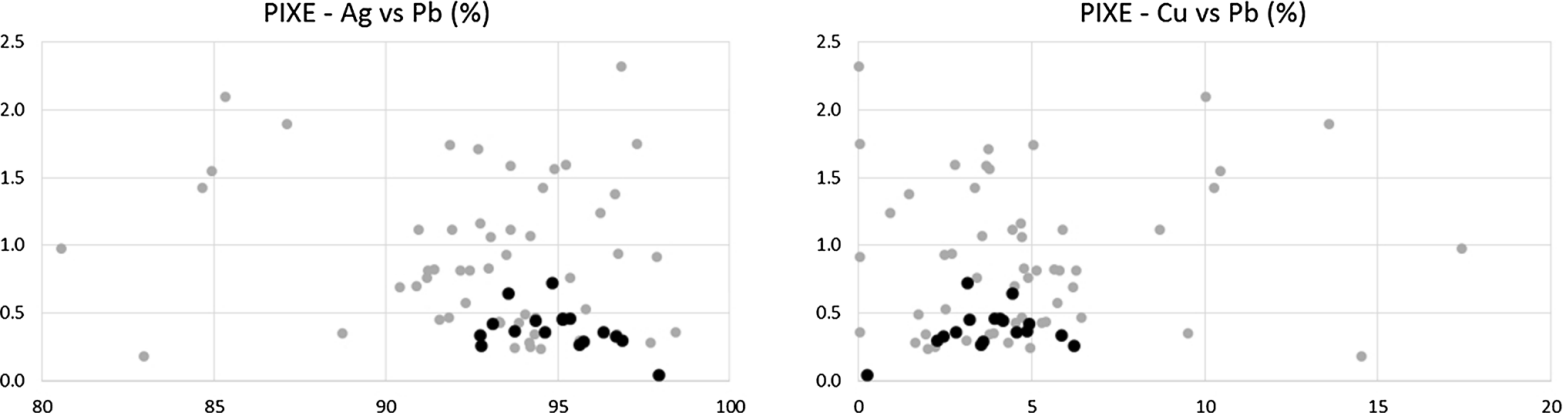
Left: Ag (horizontal) versus Pb content; Right: Cu (horizontal) versus Pb content. Black points: Mieszko II denarii, grey points: Boleslaus denarii

Figures 8 and 9 show the relationship between the three most abundant elements of the denarii, namely Ag, Cu, and Pb.

After analyzing the data the following conclusions can be drawn. The first observation is the higher elemental content definition (i.e. the narrower range of concentrations) of denarii attributed to Mieszko II (18 objects, black points, mostly later times of minting) relative to those attributed to Boleslaus (53 objects, grey points, slightly older ages). Mieszko’s denarii show generally higher Ag content and are less scattered on the phase diagrams, which suggests development and mastering of extraction and minting processes over time.

Special attention was devoted to the quantification of trace elements in the measured objects, with particular emphasis placed on Fe, Hg, Bi, Zn, and Au. The concentration values were considered reliable only if the relative error estimated by the GUPIX code was below 20%, with the strict requirement that the calculated value is higher than the calculated detection limit plus three times the estimated error (one sigma level). Data of lesser quality were ignored. Trace elements data filtered by this procedure have been compared with qualitative results of XRF measurements [3].

Trace elements can provide information about the material source. For example, the simultaneous presence of Au, Zn, and Bi indicates that the source used to extract the base metal was probably the Harz mines in Germany [11]. This combination of elements has been found in several Boleslaus denarii. These three elements were reliably quantified in 11 out of 53 denarii, while in a few cases only the Zn value was reliable, while either the Au or Bi content was doubtful. These numbers are higher than in the XRF study (only 8 denarii with Bi traces) and the difference is due to the higher sensitivity of PIXE. It is worth to note that not a single Mieszko denar was characterized by this feature and the PIXE and XRF results of this subset did not contain reliably quantified Bi traces.

Several older denarii contained very high amounts of Zn, from 0.6% up to even 2%. It was always accompanied by a very low Ag content, usually significantly below 90% and was probably reflecting the low quality of the melted alloy.

Au traces (in the range ~ 1000 to 10,000 ppm) were found in 21 denarii with only one belonging to the Mieszko subset. This observation is in agreement with XRF results, where in 19 cases (one Mieszko denar) traces of Au were detected. However, the strong correlation between Au and Ni, suggested by the XRF study, was not observed. There were only four cases that provide reliable results. In the whole collection, only 11 denarii showed reliably quantified Ni traces.

The presence of Hg is associated with a possible islamic origin [12, 13] through re-melting of Arabic denarii, abundant on Polish territory in this time. However, it is difficult to attribute this element to a single Polish ruler since good quality measurements of Hg (usually in the ~ 1000 ppm range) are present in both subsets of data (5 in Mieszko’s subset and 13 for Boleslaus). These numbers are in a very good agreement with the qualitative data obtained in the XRF study (4 and 13 denarii, respectively).

Finally, Fe was reliably quantified in all investigated objects. Its concentration covered a broad range of values from few hundreds ppm up to 2%. There was only one excessive case for which 5% was obtained. This denar exhibited a low Cu concentration below 1%. However, no significant pattern was found in these data.

## Conclusions

The combined use of micro-PIXE and micro-XRF spectrometry offers a promising and complementary approach for numismatic studies, especially under conditions when invasive methods such as cleaning, sectioning, and extraction of samples are not allowed. A reasonable match between results obtained with both techniques have been found.

Deposition of corrosion products at the surface of the denarii may be accountable for the variations observed due to the use of different micro-sampling locations with each of the two techniques. A preliminary assessment of the data suggests that the majority of the denarii analyzed have Ag surface enrichment, which is a common process observed in archaeological silver-copper alloys. A possible source of quantitative differences could be the lower penetration depth of the protons used in the PIXE analysis relative to that of the primary X-rays generated by the Rh tube used in the XRF examination. Suggested complementary techniques include NAA as a tool to gain information about the bulk material and FTIR mapping to assess the surface chemical properties.

## Electronic supplementary material

Below is the link to the electronic supplementary material.

Supplementary material 1 (DOC 765 kb)
