# Supplementary material for: Comparison of PIXE and XRF in the analysis of silver denarii of the early Piast
Source: J Radioanal Nucl Chem. 2017 Oct 20;314(3):2309–16. doi: 10.1007/s10967-017-5556-8 (PMC5707234; doi:10.1007/s10967-017-5556-8)
Supplement: Supplementary file 1 — Supplementary material 1 (DOC 765 kb) [file 10967_2017_5556_MOESM1_ESM.doc]

Supplementary information

**
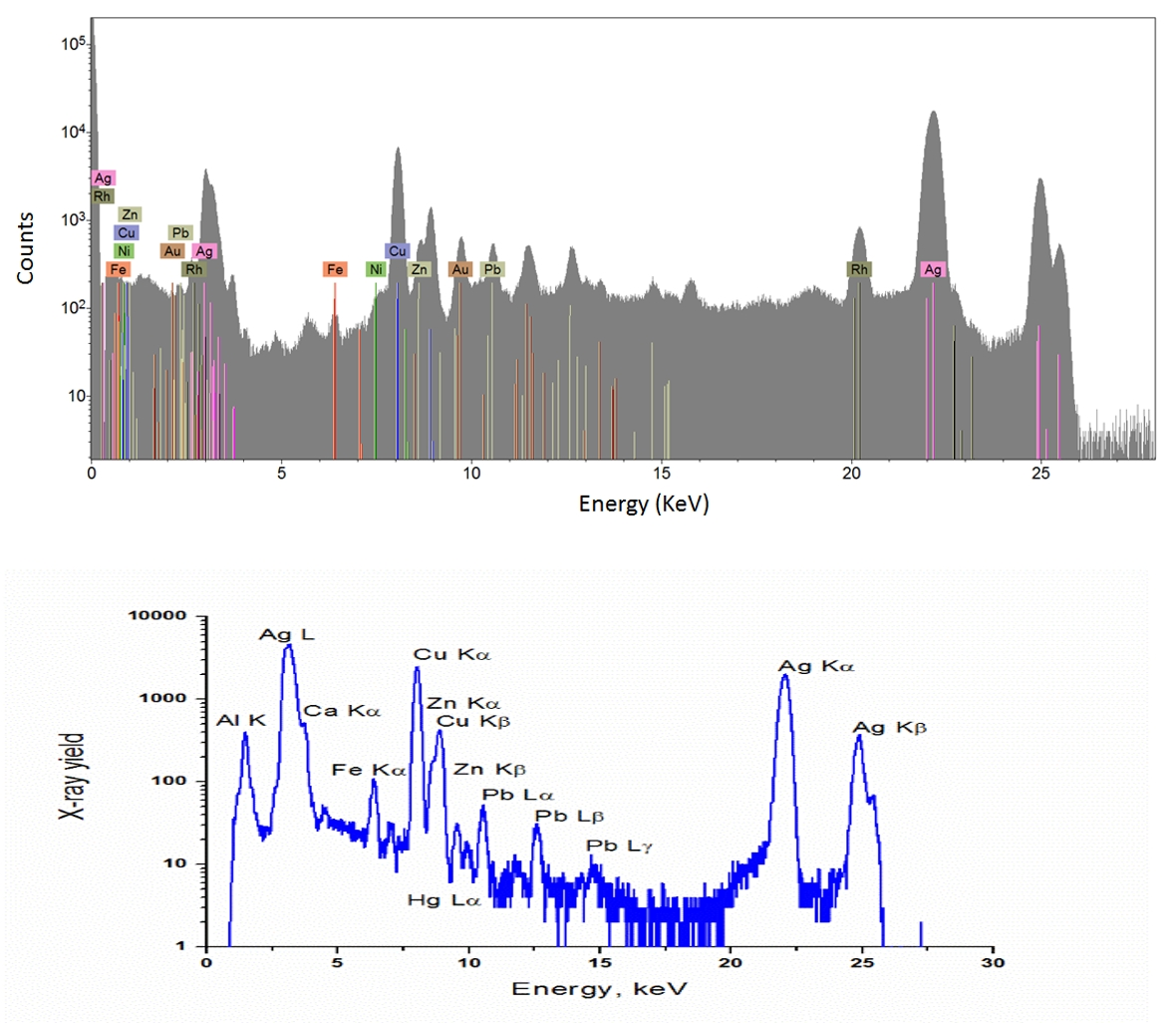
**

**Fig. 1** Comparison of typical experimental spectra obtained in XRF (top) and PIXE (bottom). The The rhodium peak present in the XRF spectrum is the excitation line.

**Fig. 2** Comparison of all data obtained for Ag and Cu using the XRF and PIXE methods. Every result is an average of 6 (PIXE) or 4 (XRF) independent measurements.

**Fig. 3** Averaged results of the multielemental PIXE measurements. Every result is an average of 6 independent measurements. Font color illustrates the averaged elemental concentration errors, with all red and magenta values treated as not reliable.
